# Participant Contributions to Person-Generated Health Data Research Using Mobile Devices: Scoping Review

**DOI:** 10.2196/51955

**Published:** 2025-01-20

**Authors:** Shanshan Song, Micaela Ashton, Rebecca Hahn Yoo, Zoljargal Lkhagvajav, Robert Wright, Debra J H Mathews, Casey Overby Taylor

**Affiliations:** 1 Biomedical Informatics & Data Science Section The Johns Hopkins University School of Medicine Baltimore, MD United States; 2 Institute for Computational Medicine Whiting School of Engineering Johns Hopkins University Baltimore, MD United States; 3 Johns Hopkins Hospital Baltimore, MD United States; 4 Department of Biomedical Engineering Johns Hopkins University Baltimore, MD United States; 5 Welch Medical Library Johns Hopkins University School of Medicine Baltimore, MD United States; 6 Berman Institute of Bioethics Johns Hopkins University Baltimore, MD United States; 7 Department of Genetic Medicine Johns Hopkins University School of Medicine Baltimore, MD United States; 8 Division of General Internal Medicine Department of Medicine Johns Hopkins University School of Medicine Baltimore, MD United States

**Keywords:** scoping review, person-generated health data, PGHD, mHealth, mobile device, smartphone, mobile phone, wearable, fitness tracker, smartwatch, BYOD, crowdsourcing, reporting deficiency

## Abstract

**Background:**

Mobile devices offer an emerging opportunity for research participants to contribute person-generated health data (PGHD). There is little guidance, however, on how to best report findings from studies leveraging those data. Thus, there is a need to characterize current reporting practices so as to better understand the potential implications for producing reproducible results.

**Objective:**

The primary objective of this scoping review was to characterize publications’ reporting practices for research that collects PGHD using mobile devices.

**Methods:**

We comprehensively searched PubMed and screened the results. Qualifying publications were classified according to 6 dimensions—1 covering key bibliographic details (for all articles) and 5 covering reporting criteria considered necessary for reproducible and responsible research (ie, “participant,” “data,” “device,” “study,” and “ethics,” for original research). For each of the 5 reporting dimensions, we also assessed reporting completeness.

**Results:**

Out of 3602 publications screened, 100 were included in this review. We observed a rapid increase in all publications from 2016 to 2021, with the largest contribution from US authors, with 1 exception, review articles. Few original research publications used crowdsourcing platforms (7%, 3/45). Among the original research publications that reported device ownership, most (75%, 21/28) reported using participant-owned devices for data collection (ie, a Bring-Your-Own-Device [BYOD] strategy). A significant deficiency in reporting completeness was observed for the “data” and “ethics” dimensions (5 reporting factors were missing in over half of the research publications). Reporting completeness for data ownership and participants’ access to data after contribution worsened over time.

**Conclusions:**

Our work depicts the reporting practices in publications about research involving PGHD from mobile devices. We found that very few papers reported crowdsourcing platforms for data collection. BYOD strategies are increasingly popular; this creates an opportunity for improved mechanisms to transfer data from device owners to researchers on crowdsourcing platforms. Given substantial reporting deficiencies, we recommend reaching a consensus on best practices for research collecting PGHD from mobile devices. Drawing from the 5 reporting dimensions in this scoping review, we share our recommendations and justifications for 9 items. These items require improved reporting to enhance data representativeness and quality and empower participants.

## Introduction

### Collecting Person-Generated Health Data for Research Using Mobile Devices and Their Facilitators

The proliferation of mobile devices boosts the generation of person-generated health data (PGHD). In 2018, more than 80% of Americans owned a smartphone [1,2], with modest growth to 85% by 2021 [3]. Wearable health care devices were used by nearly 28% of the US population by 2020 [4,5]. Among smartphone users, over 50% are collecting “health-associated information” [6]. We use the phrase “person-generated health data (PGHD)” for health-related data created, recorded, or gathered by or from individuals, family members, or other caregivers to help address a health concern inside and outside clinical settings.

PGHD from mobile devices have significant research implications. Mobile devices could facilitate access to large pools of study participant data in a granular, longitudinal, and personal way, potentially accessing high-frequency data at low costs [7]. In this study, “mobile devices” refers to smartphones and wearables, which include fitness trackers and smartwatches; “research” covers biomedical and behavior studies [8].

Because of the above advantages, researchers have made numerous efforts to collect PGHD for mobile device research. Those efforts are enabled by participants willing to share their PGHD for research [9] and by using the informatics infrastructure needed for participants to share their data. Examples of informatics infrastructure to enable PGHD sharing for research dates from 2015 and 2016, with the releases of ResearchKit (Apple Inc) [10,11] and ResearchStack (Cornell Tech and Open mHealth) [12,13] and the launch of mPower [14], Sage Bionetwork’s first major smartphone-based health research study. Research practices dealing with PGHD from mobile devices are, in part, guided by the European Union’s General Data Protection Regulation (published in 2016; in effect since 2018) and the Food and Drug Administration’s guidelines on clinical research, medical devices, and mobile health apps (released in 2013, 2015, and 2019). Between 2000 and 2020, more than 12,000 mobile device–related health research papers were published [15].

### Potential Issues With Data Representativeness and Quality When Collecting Person-Generated Health Data From Mobile Devices

Despite the potential advantages of using PGHD for mobile device research, the wide variety of practices to collect PGHD may lead to issues with data quality. These issues may be due to emerging research strategies such as Bring-Your-Own-Device (BYOD; which uses participant-owned devices instead of provisioned devices) that can miss some patient demographics [16], thus leading to low generalizability. Similar issues are noticed in studies using crowdsourcing platforms to collect data [17]. Selection bias with wearables is a significant issue due to differences in access [18,19], which in turn can lead to differences in sharing data for research [20]. Both accessibility and self-selection could lead to lower study sample diversity for race and socioeconomic status. For example, National Institutes of Health’s All of Us program allows participants to choose to contribute Fitbit (Google LLC) data through its BYOD subprogram. The All of Us BYOD subprogram has noticed a higher proportion of participants who are White, earn >US $25,000/year, and have college or advanced degrees in the BYOD subgroup compared with all participants [21]. When such data are used in machine learning, the low representation of some patient groups may lead to poor prediction accuracy when applied to those groups [22,23].

### Motivation for This Work

To better understand methodological issues such as those related to data representativeness and quality, we aimed to review publications on research collecting PGHD from mobile devices and to characterize the reporting elements of that research thoroughly, particularly those elements relevant to BYOD and crowdsourcing. In doing so, we sought to capture aspects of this field not addressed by previously published reviews. Several reviews explore mobile health research. A review by Cao et al [15] covering the years 2000 to 2020 and a review by El-Sherif et al [24] covering 2020 to February 2021 identified 4 leading contributors (the United States, the United Kingdom, Canada, and Australia). The 2 reviews, however, focused only on bibliometrics. A scoping review by Fischer and Kleen [25] published in 2021 investigated data collection by smartphone apps in longitudinal epidemiological studies, and they found a limited number of studies that integrated apps in data collection. Studies with cross-sectional designs were excluded. A scoping review by Huhn et al [26] published in 2022 summarized wearables in health research and found that most studies were observational and that 93% of the participants were in global health studies. Smartphones were not included in their scope. Other reviews of interest include one that briefly mentioned data collection using mobile devices for health research [27] and another that discussed the application of sensors (eg, inertial measurement units) in health care [28]. Our review differs from these reviews by covering wearables and smartphones and their use to collect PGHD in research. We also attempt to be comprehensive in our characterization of reporting practices, our inclusion of a range of study types (eg, both longitudinal and cross-sectional studies), and our examination of bibliographic information. To the best of our knowledge, ours is the first review of publications on research collecting PGHD from mobile devices and the first to examine BYOD practices and the use of crowdsourcing platforms in this field.

This review characterizes the reporting elements of included publications and discusses the relevance of reporting practices for supporting reproducible and responsible research. There are no current, ready-to-use guidelines for reporting research involving PGHD from mobile devices. For example, the Future of Privacy Forum’s “Best Practices for Consumer Wearables & Wellness Apps & Devices” dates back to 2016 [29]. More recently, the Office of the National Coordinator for Health Information Technology released a white paper in 2018 to conceptualize a data infrastructure for the capture, use, and sharing of PGHD in care delivery and research through 2024 [30]. The final document, however, is still under development. Also relevant are the Structured Template and Reporting Tool for Real-World Evidence (STaRT-RWE) (2021) [31] and the Mobile Health Evidence Reporting and Assessment (mERA) checklist (2016) [32], although both are limited. STaRT-RWE does not specify requirements for mobile device studies, and mERA is limited to health interventions. There are also some focused guidelines for app development (2016) [33] and data integration (2021) [34], but none fully cover what is needed for high-quality reporting of mobile device research. In fact, reporting deficiencies are widely noted in studies involving mobile devices. A systematic review by Olaye et al [35] in 2022 found that 30% of publications failed to report quantitative adherence; such missingness can impede a complete understanding of study data. Our review characterizes the magnitude and types of reporting problems in publications on research collecting PGHD from mobile devices.

## Methods

We performed a scoping review following the PRISMA-ScR (Preferred Reporting Items for Systematic Reviews and Meta-Analyses extension for Scoping Reviews) checklist (Multimedia Appendix 1) [36]. The scoping review first involved implementing a comprehensive search and screening strategy. Next, the selected articles were characterized according to their bibliographic details and 5 reporting dimensions.

### Search Strategy

A PubMed search was used to find instances of explicitly described participant data contributions (eg, through the use of language such as “data donation,” “data sharing,” etc) involving wearables, smartphones, or mobile apps (Textbox 1). A MeSH (Medical Subject Headings) term analysis of a preliminary sample of articles was conducted to support PubMed query development (Yale MeSH Analyzer [37]). We selected search terms to balance recall and precision in the search results. We ran the PubMed search on August 7, 2021.

**PubMed search strategy.**

- Search concepts
- Research questions:
  - Q1a. Data donation
    - (data[tw] OR record*[tw] OR information[tw]) AND (donat*[tw] OR donor*[tw])
  - Q1b. Data sharing
    - (data[tw] OR record*[tw] OR information[tw]) AND (“Information Dissemination”[Mesh] OR sharing*[tw] OR share*[tw])
  - Q2. Wearables, smartphones, or mobile applications
    - “Wearable Electronic Devices”[Mesh:NoExp] OR “Fitness Trackers”[Mesh] OR wearable sensor*[tw] OR wearable device*[tw] OR wearable technolog*[tw] OR self-tracking[tw] OR self-tracker*[tw] OR fitness tracker*[tw] OR smart watch*[tw] OR smartwatch*[tw] OR “Mobile Applications”[Mesh] OR “Cell Phone”[Mesh:NoExp] OR “Smartphone”[Mesh] OR “Computers, Handheld”[Mesh] OR Mobile health[tw] OR mHealth[tw] OR eHealth[tw] OR e-health[tw] OR e-healthcare[tw] OR mobile application*[tw] OR mobile technolog*[tw] OR app[tw] OR apps[tw] OR cell phone*[tw] OR cellphone*[tw] OR cellular phone*[tw] OR cellular telephone*[tw] OR mobile phone*[tw] OR mobile telephone*[tw] OR smart phone*[tw] OR smartphone*[tw] OR mobile device*[tw] OR personal digital assistant*[tw] OR “Digital Technology”[Mesh] OR digital technolog*[tw]
- Full search strategy
- (Q1a OR Q1b) AND Q2

### Screening and Data Charting

Covidence [38] was used to screen publications for eligibility in 2 steps, first by eliminating publications based on a title and abstract review and then by reviewing the full text of the remaining journals. A pre-established set of eligibility criteria was used for screening (Table 1). As this an emerging field and our motivation to see the trends in full landscape, we did not set criteria for time range. Screening decisions were made only after 2 independent reviewers on the screening team (SS, MA, and RHY) agreed. All conflicts were resolved by group discussion. Library journal subscriptions and interlibrary loan were used to obtain full text, with the full text of 1 publication [39] not available from either source. All reviewers conducted pilots for each round of screening.

We charted publications for bibliographic details and along 5 reporting dimensions (“participant,” “device,” “data,” “study,” and “ethics”; double quoted here and in the text below). Because our focus is on health data contributions from participants through BYOD and crowdsourcing platforms, we modeled the reporting dimensions on existing work in the areas of citizen science data contribution [40], bioethical approaches to using personal health data [41], and mobile health apps [42]. Upon reviewing the keywords of included publications (eg, “ethical code,” “consent,” and “informed consent”), we summarized frequent themes of keywords (eg, consent). Furthermore, we refined our charting template by selecting factors related to those frequent themes to improve data adequacy. The final factors included under the 5 reporting dimensions are listed in Table 2. A data charting pilot with a random sample of one-fifth of the final pool of publications was conducted before finalizing the data charting template [43]. A team of 4 reviewers (SS, MA, RHY, and ZL) charted the data using the template built in Covidence [38]. In addition, 2 reviewers independently charted each publication. Consensus was reached by group discussion.

**Table 1:** Inclusion and exclusion criteria overview.

Theme	Criteria
Phenomenon of interest	Data contribution (ie, data donation or data sharing).
Technology	Wearables, smartphones, or mobile applications: limit the scope of wearables to fitness trackers and smartwatches and include surveys through smartphone applications. Exclude surveys through websites.
Perspective	Individual: individual refers to either patient or nonpatient and exclude data transfer between researcher and researcher, researcher and organization, and organization and organization.
Goal	Collected data is used for research.
Data Type	Data concerning human health.
Study Type	—^a^
Language	English.
Time	—

^a^Not applicable.

**Table 2:** Definitions of 24 factors under bibliographic details and 5 reporting dimensions.

Factors					Definitions
**Bibliographic details (3 factors)**					
		Publication year			The year of publication, not the year of submission or acceptance.
		Publication location			The country or area of the affiliation of the first author or first authors.
		Publication type			Research: original research publication. Protocol: protocol or proposal for original research. Tech: publication describing a dataset, database, mobile app design, platform, or algorithm; or use case report. Review: systematic review, scoping review, or any other type of review. Other: including viewpoint, perspective, editorial, commentary, correspondence, letter, or needs assessment; mostly about ethics and regulations.
**Reporting dimensions (21 factors)**					
	“**Participant”**				
			Identity of data subject and data contributor	This factor examines the question, “Are the data subject and data contributor the same individual?” Participant in a study could be either data subject or data contributor. Data subject refers to the person whose data is collected, held, or processed. Data contributor refers to the individual who provides information for a research study. Data contributor may contribute data on behalf of data subject. Data contributors may include, but are not limited to, guardians of children, health care providers of patients, and sheriffs or jail administrators of prisoners. Identity refers to whether the data subject and data contributor are the same individual.	
			Living status of data subject	Whether data subject is living or not at the moment of data contribution to research.	
			Purpose of data generation	Whether the data contributed to research were generated particularly for this research or generated for other purposes and then used for research. If data were generated for participants themselves during routine use (eg, self-management), this is an example of “other purposes,” and it is marked as “for self.”	
	“**Device”**				
			Device owner	Owner of the device used to generate the data, not the owner of device used to transfer the data, although the 2 devices could be the same.	
			Device type	Wearable devices include fitness trackers and smartwatches.	
			Data capture mode	Passive or active.	
	“**Data”**				
			Data type	Location data is any type of data revealing location, which may include, but is not limited to, GPS data and self-reported location data. Genetics-biospecimen data may include, but is not limited to, genetics data extracted from biospecimens. Genetics-non-biospecimen data may include, but is not limited to, genetics data not extracted from biospecimens; for example, data from commercial genetics tests. Claims or administrative data may include, but is not limited to, insurance claims data and administrative data such as length of stay, date of service, etc. Clinical data may include, but is not limited to, electronic health records (eg, laboratory results, vital signs, and treatments), health surveys, clinical trial data, and patient or disease registries. Mental health or lifestyle data are those not included in the categories above. They may include data relevant to, but not limited to, mental health, diet, physical activity, or sleep.	
			Data owner	Data ownership is charted based on explicit statements in the text of publications.	
			Participant access to data after contribution	Whether participants can access contributed data after data contribution.	
	“**Study”**				
			Research scenario	Nonprofit, commercial, or public health.	
			Purpose of data collection	For primary, secondary, or both primary and secondary data analysis.	
			Used crowdsourcing platform to collect data	In our review, we focused on crowdsourcing platforms for data collection. These platforms have 3 essential properties, that are researchers can access potential participants through the platform, researchers can distribute tasks to participants through the platform, and the number of potential participants and enrolled participants is large. Platform examples include Amazon Mechanical Turk and PatientsLikeMe (PatientsLikeMe website).	
			Research design	Observational or experimental.	
			Design duration	Cross-sectional or longitudinal, not restricted to observational studies.	
			Attrition reported	Whether the publication reported attrition or not. In our review, we define attrition as “the loss of eligible participants from research at any time following consent to participate” (adapted from Siddiqi et al [44]).	
	“**Ethics”**				
			Consent reported	Whether the publication reported consent or not.	
			Informed consent reported	Whether the publication reported informed consent or not.	
			Consent type	Consent type is charted based on explicit statements in the publication’s text or on the reviewer’s best assumptions according to details mentioned in the publication. Blanket consent: a process by which participants contribute their data without any restrictions [45]. Broad consent: a process by which participants contribute their data, subject to specified restrictions, for multiple future studies in a broad range, the nature and specificities of which are unknown at the time of consenting [45,46]. Tiered consent: a process by which participants have the option of giving broad consent for only certain types of research or research uses, including for only specific diseases or indications (eg, neurological diseases), for only publicly funded research, or for only specified institutions or researchers. With tiered consent, participants can also choose whether their data are identifiable or anonymized [47]. Meta consent: a form of tiered informed consent in which participants can choose a different consent option within different research tiers. For example, a participant could give broad consent to all research conducted in certain institutions but only study-specific consent for genomic research or privately funded research [47]. Suppose a study reports both meta-consent and dynamic consent^a^. In that case, this study will be marked as “meta consent” because dynamic consent is considered to be a characteristic of meta consent by some, but not all, researchers. However, if a study only reports dynamic consent, this study will not be marked as “meta consent” or any other consent because dynamic consent itself is not a consent model [46]. Explicit consent: a process in which the data subject must give an express statement of consent. An explicit consent statement should specifically refer to (1) the particular dataset that is to be processed, (2) the precise purpose of processing (including any automated decision-making), (3) any risks or implications that might arise for the data subject as a result of the data processing, and (4) any other relevant and specific information that might influence the decision of a data subject to give or not give their consent [48]. Here, explicit consent is used interchangeably with study-specific consent. With study-specific informed consent, research participants consent to their contributed data being used in a single specific research study. If researchers choose this form of consent, they will need to contact data contributors whenever they want to use a particular data point in a new study [47].	
			Consent subject	Whether or not the publication reported consent subjects. Consent subjects are individuals who are consented to participate in research studies.	
			Right to opt-out reported	Whether or not the publication mentioned the participants’ right to opt out of research at any time.	
			Monetary benefits	“Unconditional” refers to scenarios where participants can receive monetary benefits as long as they participate, no matter their responses or engagement. “Conditional” refers to scenarios where participants can receive monetary benefits if they satisfy specific criteria after participation.	

^a^Dynamic consent is a process facilitated by collaborative and online digital platforms that allows participants to regularly check research activities and modify their consent for any upcoming research projects [46,47]. Dynamic consent can also be described as an approach to consent that enables ongoing engagement and communication between individuals and the users and custodians of their data [49].

### Data Analysis and Visualization

To determine the leading countries and their contributions to various publication types, we compared the counts and percentages of publications by type and location. In addition, we visualized the number of publications by year to evaluate publication trends. We also visualized publication counts by year and location for original research publications. Reporting dimensions, including factors concerning “participant,” “device,” “data,” “study,” and “ethics” (Table 2), were analyzed for original research publications only. We charted the frequency and percentage of original research studies that included these 21 reporting dimension factors.

In addition, 4 factors with multiple-choice options were visualized using Venn (if options ≤3) or alluvial (if options >3) diagrams after excluding publications that did not report these factors. These 4 factors included 1 “device” dimension factor (device owner), 2 “data” dimension factors (data type and data owner), and 1 “study” dimension factor (research scenario). We also analyzed publications according to the frequency of data types collected. We assessed missing factors among original research publications to evaluate changes in reporting deficiencies for each reporting dimension. We compared the percentages of publications missing factors between 2 temporal groups (2010-2016 and 2017-2021). All analyses and visualizations were performed using R (version 4.2.1; R Core Team).

## Results

### Search and Data Charting

**Figure 1:**
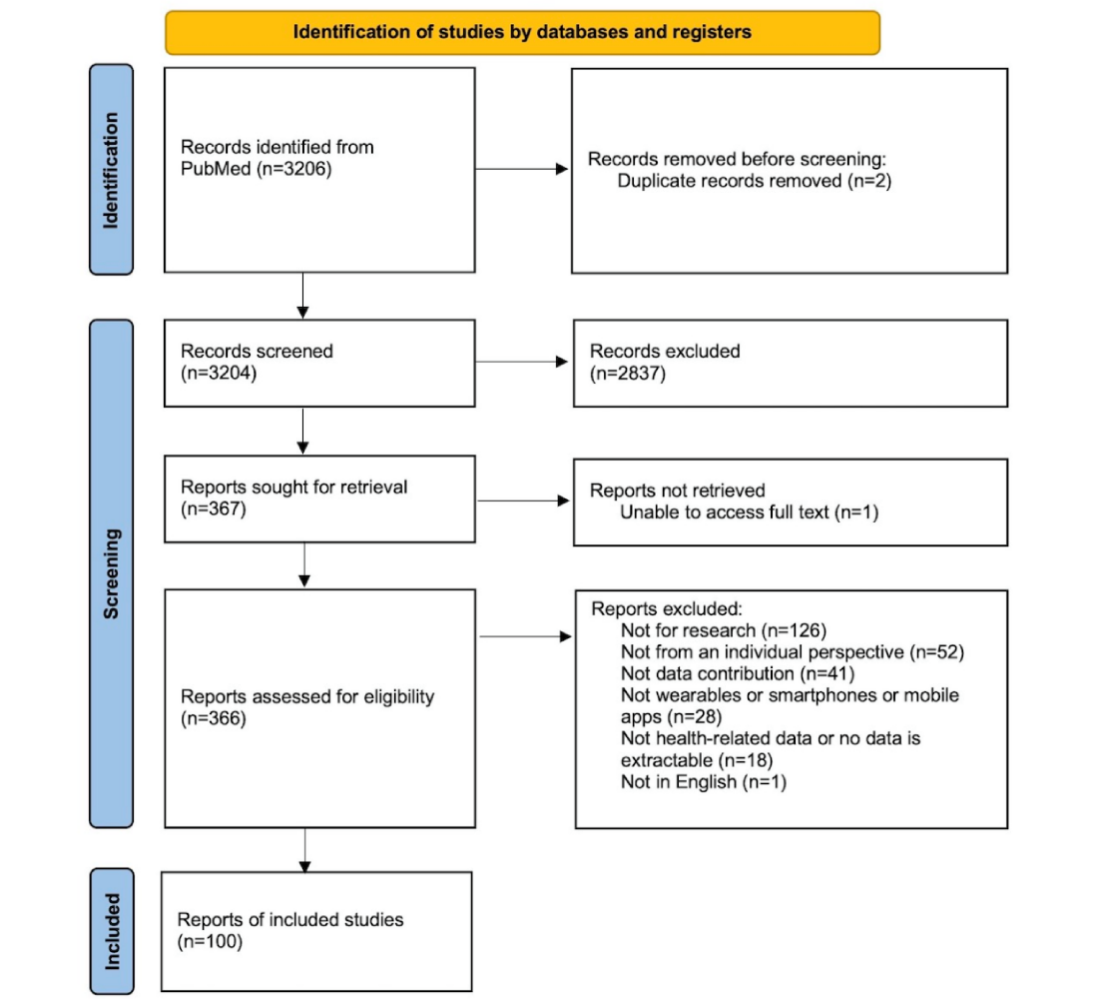
PRISMA-ScR (Preferred Reporting Items for Systematic Reviews and Meta-Analyses extension for Scoping reviews) flow diagram.

Our search strategy yielded 3206 results. A total of 100 publications remained following screening [50-149]. All 100 articles are included in our data analyses (Figure 1; bibliographic information in Multimedia Appendix 2).

### Trends by Publication Year, Location, and Publication Type

**Figure 2:**
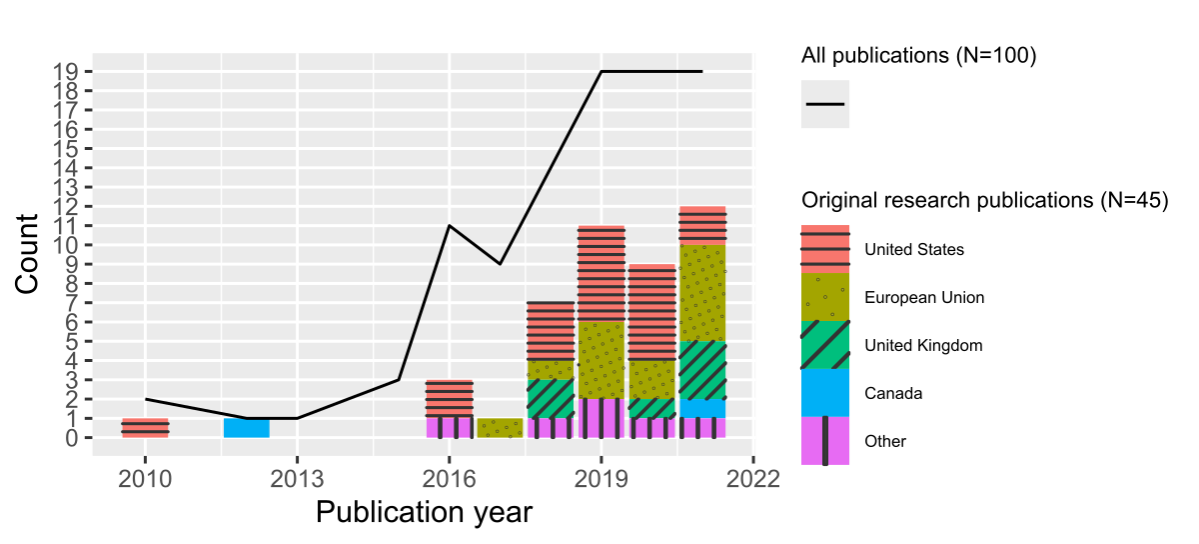
Bibliographic details of all publications by year and original research publications by year and location (data from January 2010 to August 2021).

Publication year and location were extracted from the 100 included articles and organized by publication type (Figure 2, Multimedia Appendix 3). The majority of publications had a first author from the United States (46%, 46/100), European Union (20%, 20/100), United Kingdom (11%, 11/100), or Canada (6%, 6/100). Publications designated as “Research” (original research) made up the largest proportion of publications (45%, 45/100). These were followed by publications designated as “Tech” (20%, 20/100), “Other” (17%, 17/100), “Review” (13%, 13/100), and “Protocol” (5%, 5/100). Refer to Table 2 for definitions of these publication types.

There was a spike in total publications in 2016, with 11 publications in 2016 compared with 3 or fewer annually beforehand. Since 2016, a minimum of 9 publications have been published annually. There was another spike in output in 2019, with 19 publications. Other trends observed were US-dominant “Tech” publications and diversely geolocated “Review” publications.

### Reporting in Original Research Publications

#### Reporting of Participant Factors

Most publications (78%, 35/45) declared or implied that the data subject and contributor were the same individual. Almost all data subjects (96%, 43/45) were living at the time of data contribution. In most publications, analyses were conducted using data generated by data subjects for themselves during routine use (56%, 25/45) rather than explicitly for research (11%, 5/45; Table 3).

**Table 3:** Counts and percentages of original research publications along with reporting factors.

Reporting factors				Original research publications (N=45), n (%)
“**Participant” dimension**				
	**Identity of data subject and data contributor** **(Are the data subject and the data contributor the same individual?)**			
		Yes	35 (78)	
		No	0 (0)	
		Mixed	6 (13)	
		Not available	4 (8)	
	**Living status of the data subject**			
		Living	43 (96)	
		Both living and deceased	1 (2)	
		Not available	1 (2)	
	**Purpose of data generation**			
		For research	5 (11)	
		For self	25 (56)	
		Not available	15 (33)	
“**Device” Dimension**				
	**Device owner (multiple choices possible)^a^**			
		Data subject	19 (42)	
		Data contributor	18 (40)	
		Researcher	9 (20)	
		Not available	17 (38)	
	**Device type**			
		Wearable	7 (16)	
		Smartphone	17 (38)	
		Both	20 (44)	
		Not available	1 (2)	
	**Data capture mode**			
		Active	8 (18)	
		Passive	8 (18)	
		Both	22 (49)	
		Not available	7 (16)	
“**Data” Dimension**				
	**Data type (multiple choices possible)^a^**			
		Location	18 (40)	
		Genetics, biospecimen	2 (4)	
		Genetics, non-biospecimen	3 (7)	
		Claims or administrative	1 (2)	
		Clinical	25 (56)	
		Mental health or lifestyle	38 (84)	
	**Data owner (multiple choices possible)^a^**			
		Data subject	3 (7)	
		Data contributor	4 (9)	
		Researcher	0 (0)	
		Company	3 (7)	
		The public	0 (0)	
		Not available	41 (91)	
“**Study” Dimension**				
	**Participant access to data after contribution**			
		Yes	7 (16)	
		No	0 (0)	
		Not available	38 (84)	
	**Research scenario (multiple choices possible)^a^**			
		Public health	10 (22)	
		Commercial	16 (36)	
		Nonprofit	37 (82)	
		Not available	2 (5)	
	**Purpose of data collection**			
		Primary	18 (40)	
		Secondary	0 (0)	
		Both	12 (27)	
		Not available	15 (33)	
	**Used crowdsourcing platform to collect data**			
		Yes	3 (7)	
	**Research design**			
		Observational	41 (91)	
		Experimental	4 (9)	
	**Design duration**			
		Cross-sectional	25 (56)	
		Longitudinal	18 (40)	
		Not available	2 (4)	
	**Attrition reported**			
		Yes	14 (31)	
“**Ethics” Dimension**				
	**Consent reported**			
		Yes	41 (91)	
	**Informed consent reported**			
		Yes	28 (62)	
	**Consent type**			
		Blanket	0 (0)	
		Broad	2 (4)	
		Tiered	1 (2)	
		Meta	0 (0)	
		Explicit	5 (11)	
		More than one type of consent	2 (4)	
		Not available	35 (78)	
	**Consent subject**			
		Data subject	4 (9)	
		Data contributor	4 (9)	
		Both	29 (64)	
		Not available	8 (18)	
	**Right to opt-out reported**			
		Yes	9 (20)	
	**Monetary benefits**			
		Unconditional	2 (4)	
		Conditional	9 (20)	
		No benefit	6 (13)	
		Not available	28 (62)	

^a^Levels below reporting factors were cleaned to be binary if multiple choices were possible.

#### Reporting of Device Factors

**Figure 3:**
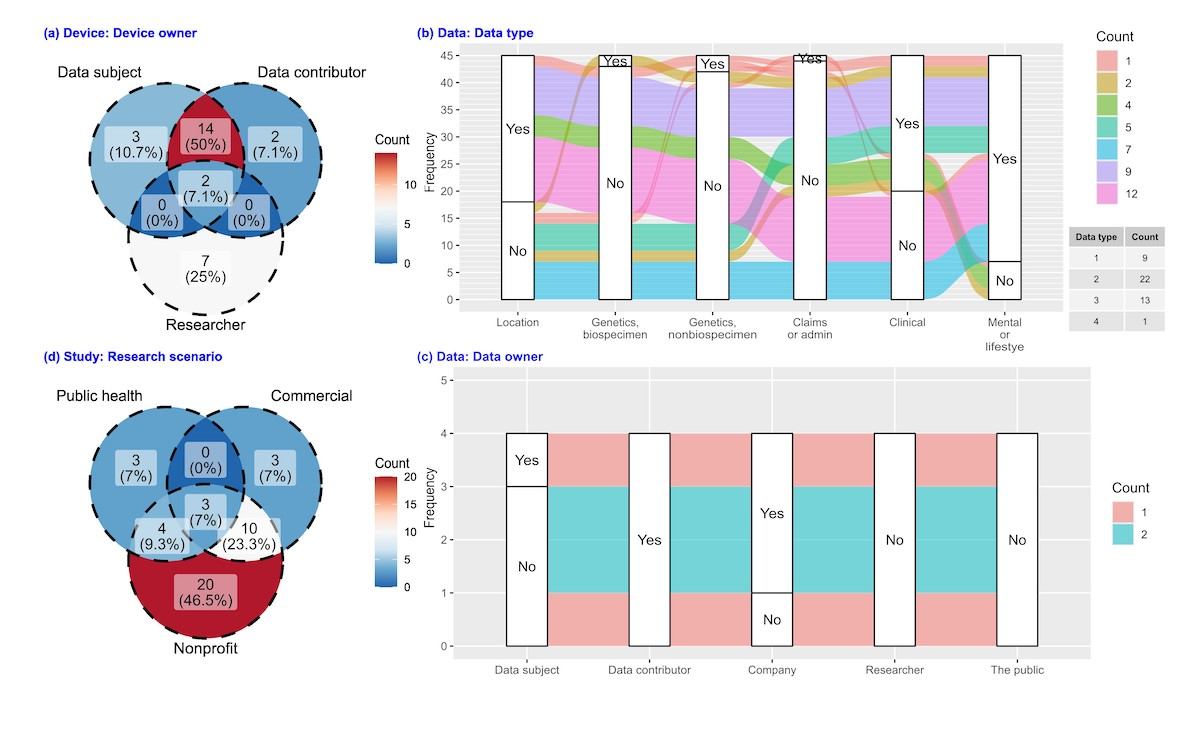
Publication counts for 4 reporting factors with multiple choices possible, excluding publications that did not report these factors, (A) device owner, (B) data type, (C) data owner, and (D) research scenario. The data type figure includes a table presenting publication counts by the number of reported data types.

Among publications reporting the device owner, more reported that devices were owned by participants (data subject or data contributor) than were owned by researchers (75%, 21/28 vs 32%, 9/28; Figure 3A). Furthermore, 2 publications reported a mix of device ownership (owned by data subject, data contributor, and researcher), indicating a transfer of ownership (ie, participants retained the provisioned device after the study). Wearables were more often used with smartphones than used alone (wearables and smartphones 44%, 20/45 vs wearables alone 16%, 7/45; Table 3). Most publications reported a combination of active and passive data capturing, and active-alone and passive-alone data capturing were equally popular (active and passive 49%, 22/45 vs active-alone 18%, 8/45 vs passive-alone 18%, 8/45; Table 3).

#### Reporting of Data Factors

With multiple choices possible, the mental health or lifestyle data type (84%, 38/45; Table 3) was the most collected, followed by clinical (56%, 25/45) and location (40%, 18/45) data types. In comparison, genetics data types (biospecimen 4%, 2/45 and nonbiospecimen 7%, 3/45 [Table 3]; in total 11%, 5/45 [Figure 3B]) and insurance claims or administrative data type (2%, 1/45; Table 3) were seldom collected. Most publications reported more than one data type (80%, 36/45; refer to table in Figure 3B). Overall, 40% (18/45) of publications’ collected data included clinical data and mental health or lifestyle data (Figure 3B). No dominant data ownership arrangement was observed (data subject 7%, 3/45 vs data contributor 9%, 4/45 vs company 7%, 3/45; Table 3).

#### Reporting of Study Factors

Most studies that reported research scenarios were conducted within a strictly nonprofit milieu (47%, 20/43; Figure 3D). No study collected data solely for secondary analysis purposes (Table 3). A limited number of publications reported using crowdsourcing platforms to collect data (7%, 3/45; Table 3). Most studies were observational (91%, 41/45; Table 3). A prevalence of cross-sectional designs over longitudinal designs was observed (56%, 25/45 vs 40%, 18/45; Table 3).

#### Reporting of Ethics Factors

No publication reported blanket or meta-consent (Table 3). Among those publications reporting monetary benefits for participation, more than one-third reported giving no monetary benefits to participants (35%, 6/17; Table 3).

#### Reporting Deficiencies in Original Research Publications

**Figure 4:**
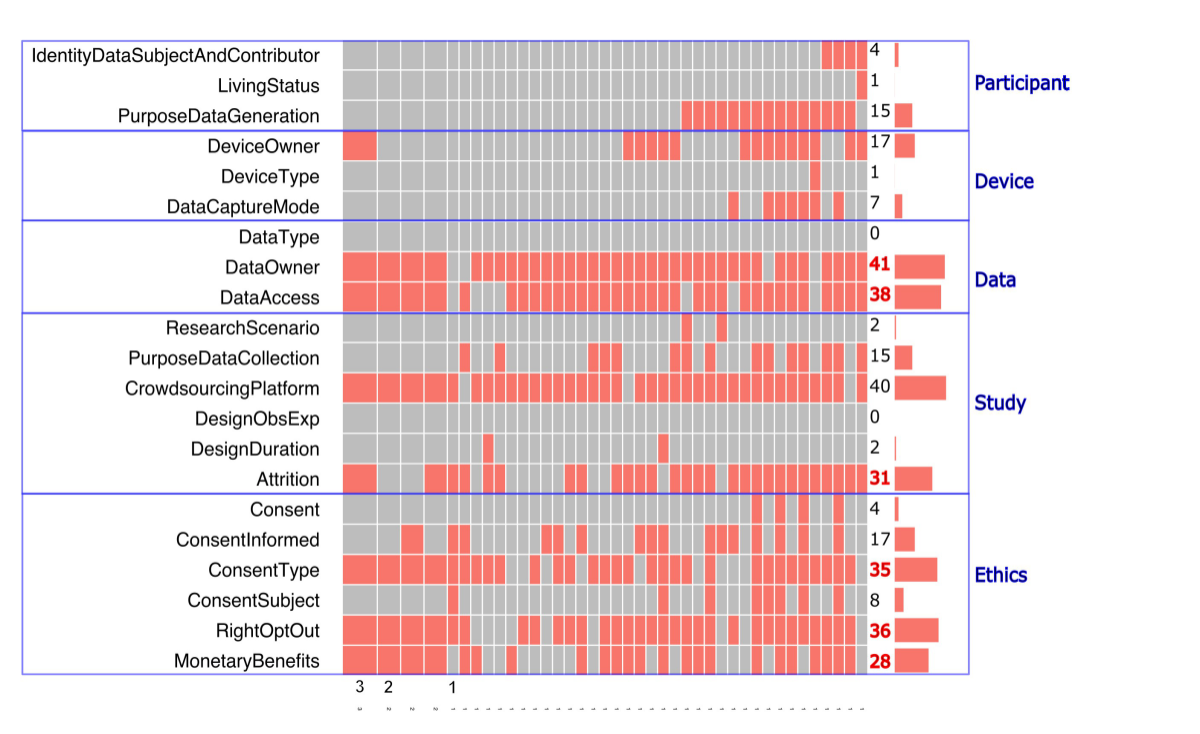
Missingness in 21 reporting factors in original research publications (N=45). The red color represents instances of missingness. Publication counts on the right vertical axis are highlighted in red if missingness existed in more than half of the publications for a particular reporting factor (excluding the factor “CrowdsourcingPlatform” for those that used crowdsourcing platforms to collect data). For a definition of each reporting factor, refer to Table 3.

The number of publications that missed reporting at least 1 factor under the 5 reporting dimensions broke down as follows: 36% (16/45) for the “participant” dimension, 42% (19/45) for the “device” dimension, 96% (43/45) for the “data” dimension, 78% (35/45) for the “study” dimension, and 96% (43/45) for the “ethics” dimension (Figure 4).

Except for the data type factor (“DataType”) and the observational or experimental study design factor (“DesignObsExp”), missingness existed in most factors under the 5 reporting dimensions. In total, 6 factors had missingness in more than half of the original research publications: data owner (91%, 41/45), participant access to data after data contribution (84%, 38/45), right to opt out (80%, 36/45), consent type (78%, 35/45), monetary benefits (62%, 28/45), and attrition (69%, 31/45). These 6 factors were often missing together (Figure 4).

The factor for marking studies that used crowdsourcing platforms to collect data (“CrowdsourcingPlatform”) was excluded from the reporting deficiency analysis because using these platforms is optional for research.

**Figure 5:**
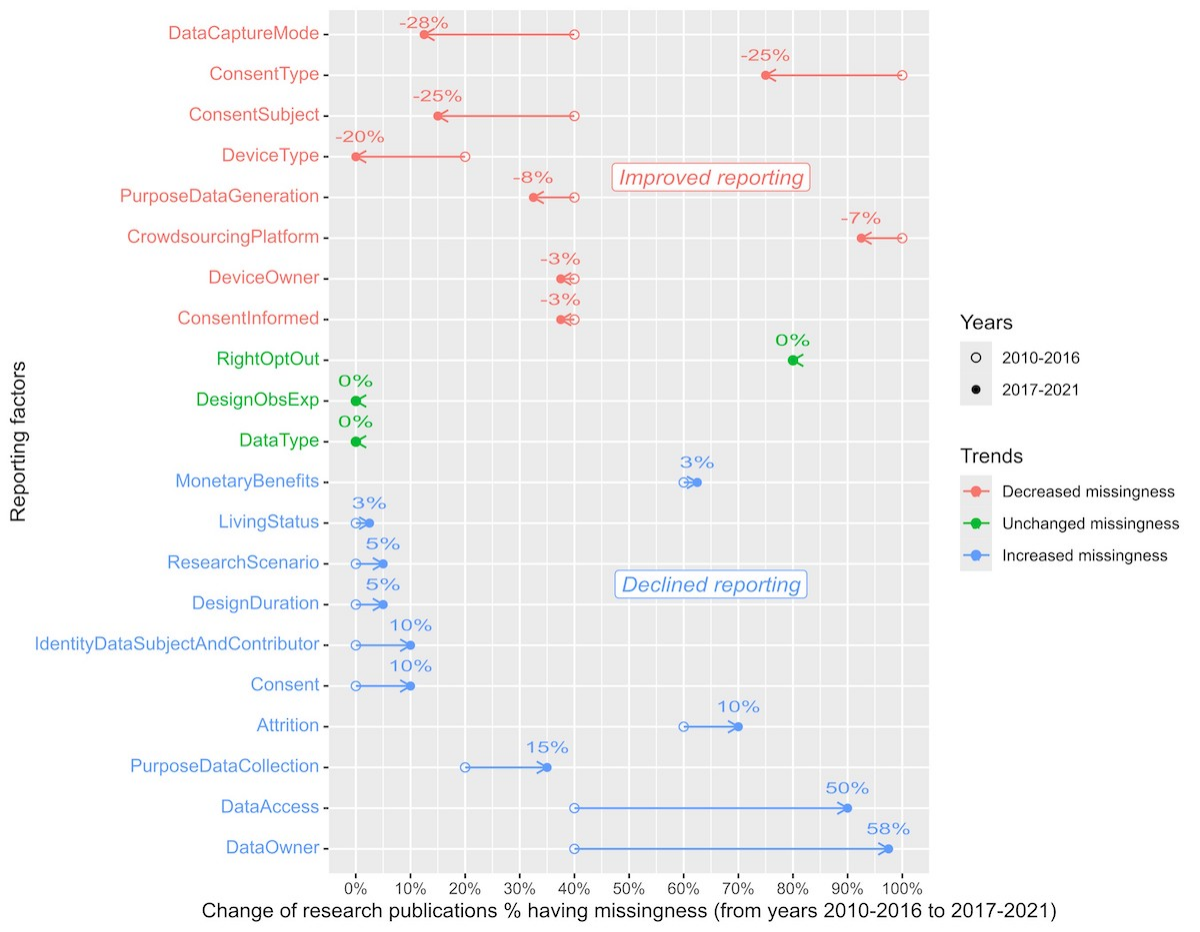
Shift in the reporting deficiencies of original research publications, measured by the % of missingness across 21 factors between 2010-2016 and 2017-2021 (sorted by % change). For a definition of each reporting factor, refer to Table 3.

Trends in reporting deficiencies were observed in original research publications over time. Reporting deficiencies were measured by the absence, or missingness, of specific reporting factors, with increased missingness reflecting declining reporting performance and decreased missingness reflecting improving reporting performance. The reporting performance of some reporting factors showed declines and others improvements when compared across the years 2010-2016 and 2017-2021. Furthermore, 2 factors in the “data” dimension exhibited substantial declines in reporting performance: data owner (58% change in missingness; from 2/5, 40% in 2010-2016 to 39/40, 98% in 2017-2021) and participant access to data after contribution (50% change in missingness; from 2/5, 40% in 2010-2016 to 36/40, 90% in 2017-2021). In contrast, 4 factors in the “device” and “ethics” dimensions exhibited substantial improvements in reporting performance: data capture mode (–28% change in missingness; from 2/5, 40% in 2010-2016 to 5/40, 13% in 2017-2021), device type (–20% change in missingness; from 1/5, 20% in 2010-2016 to 0/40, 0% in 2017-2021), consent type (–25% change in missingness; from 5/5, 100% in 2010-2016 to 30/40, 75% in 2017-2021), and consent subject (–25% change in missingness; from 2/5, 40% in 2010-2016 to 6/40, 15% in 2017-2021). The magnitude of change in the percentage of publications with missingness was substantial if the absolute value of the difference was equal to or greater than 20% (Figure 5 and Multimedia Appendix 4).

## Discussion

### Principal Results

We have characterized the reporting practices of published research studies that collected PGHD using mobile devices. Our findings show that the United States has gained momentum in publishing in this area over the past decade (Figure 2). In addition, a high proportion of the original research articles we identified used participant-owned devices or a BYOD strategy if they reported device ownership (75%, 21/28; Figure 3A), with few studies collecting data using crowdsourcing platforms (7%, 3/45; “Used crowdsourcing platform to collect data” in Table 3). We also found reporting deficiencies in the “data” and “ethics” dimensions among original research articles, where most original research articles were deficient in at least 1 factor concerning “data” (96%, 43/45) or “ethics” (96%, 43/45; Figure 4).

#### Trends by Publication Year, Location, and Publication Type

Our analysis of publications by year and location echoed trends seen in the field, with publication spikes in 2016 and 2019 and stable development afterward (Figure 2). Like others [15,24], we found that the United States is a leading contributor to these publications. Furthermore, the United States dominates contributions in several publication types, including original research (Multimedia Appendix 3).

#### Reporting in Original Research Publications

In the “participant” reporting dimension, we found that the mobile device data used for research were more often generated by participants for themselves through routine use rather than generated explicitly for research (56%, 25/45 vs 11%, 5/45; “Purpose of data generation” in Table 3). However, the quality of data acquired for routine use may not meet data quality requirements for research. For instance, individuals engaging in routine device use may not be trained in the best way to wear and use the device to ensure high-quality data collection, thereby leading to potential issues with low or missing wear time and user errors that impact the completeness and correctness of the collected data [150]. Systematic data quality assessment tools have been developed specifically for the secondary use of PGHD collected from mobile devices. These tools address the quality issues of routine use data by recommending research reporting clarity on the definition of nonwear time, the threshold for valid records, the definition of data completeness, and the identification of outliers [151].

In the “device” reporting dimension, among publications that reported device ownership, we found that three-quarters of original research publications (21/28; Figure 3A) reported using mobile devices owned by participants (data contributors or data subjects), which implies the popularity of the BYOD strategy. As mentioned in the introduction, BYOD may bring bias into demographics through disparate accessibility to device and self-selection, thus a comprehensive guideline on using BYOD to collect PGHD, which is not available so far, is a need. As an alternative to BYOD, some researchers provide devices to participants (9/28; Figure 3A). In our review, for studies that provided devices to participants, participants in 2 of them (Figure 3A) retained the device after the study (shown in Figure 3A as a mix of device ownership). This strategy may serve as an incentive and help improve device use simultaneously. In our own research [152], however, we found that providing a device when recruiting study participants for cardiovascular wearable studies did not improve sample representativeness in age, race, and education. Thus, more work is needed to understand effective incentives to participate in research that collects PGHD from mobile devices such that recruitment would lead to a more representative study sample.

We found that wearables were predominantly used as secondary devices connected to smartphones rather than as standalone devices (44%, 20/45 vs 16%, 7/45; “Device type” in Table 3), indicating that barriers may exist to the direct collection of data from wearables for research purposes. Evidence to support this supposition is provided by a study published in 2023 [153] that reported that among those who expressed a willingness to share data from a wearable, only 25% deposited their data in the study’s research database. The primary reason for the refusal to deposit data was the inconvenience of the data transfer process. Previous research findings [154] have also raised concerns that wearables may be less sustainable than smartphones for remote monitoring over long periods [155]. However, the reliance on a smartphone for data transfer is not ideal if other data are not collected by the smartphone, such as information about heart rate or sleep. A review published in 2022 [156] found that a limited number of mobile health apps use Bluetooth, and an even smaller number use standard Bluetooth Low Energy. This indicates a potentially low capability of smartphone apps to interact with external devices, including different types of wearables.

Researchers adopt mobile devices for research due to their ability to capture data passively, thus reducing recall bias introduced by active approaches (eg, surveys). Our findings, however, suggest that active and passive data capturing were equally popular (18%, 8/45 for each; “Data capture mode” in Table 3) and frequently used in tandem (49%, 22/45; “Data capture mode” in Table 3). Researchers may have hesitated to depend solely on passive data collection because this approach can result in low participant engagement due to privacy concerns [157] and potential data quality issues [150]. An approach that uses both passive and active data collection can offer more comprehensive data and enable cross-validation of responses through data linkages.

In the “data” reporting dimension, a majority of the publications reported collecting multiple data types (80%, 36/45; refer to table in Figure 3B). Furthermore, 40% (18/45) of publications’ collected data include clinical data and mental health or lifestyle data (Figure 3B). A subset of these publications indicated that clinical data were collected from participants’ electronic health records, reflecting progress made to integrate mobile device data into electronic health records [158]. Studies seeking to connect multiple data types from study participants should exercise caution due to the increased risk of participant reidentification [6].

We found that certain data types were more infrequently collected than others. For example, insurance claim and administrative data were rarely collected (2%, 1/45; “Data type” in Table 3). This gap may be because of difficulties linking these data to other data [159]. Also, despite advancements in informatics infrastructure (eg, the HL7 FHIR [Fast Health Interoperability Resources] API) enabling patients to download these data (eg, Medicare claims and encounter data through Blue Button [160]), only a limited number of beneficiaries have done so. For example, while Blue Button, available since 2010, has been used by over one million Medicare beneficiaries [161], this is a small fraction of the 65 million people covered by Medicare as of 2022 [162]. Genetics data collection was also rare (11%, 5/45; Figure 3B), which is consistent with the prevailing observation that a limited number of genomics mobile apps serve to collect data, compared with other tasks (eg, education and communication) [163,164].

In the “study” reporting dimension, only 7% of original research publications used crowdsourcing platforms to collect data (3/45; “Used crowdsourcing platform to collect data” in Table 3), which may indicate a scarcity of streamlined mechanisms for using these platforms. Given the growing popularity of BYOD research, there is an opportunity for improved mechanisms to leverage such platforms to transfer data from device owners to researchers. We also found that the majority of the studies were observational (91%, 41/45; “Research design” in Table 3), aligning with previous research [26]. Although mobile devices enable facile longitudinal data collection, we discovered a slightly greater prevalence of cross-sectional studies over longitudinal studies (56%, 25/45 vs 40%, 18/45; “Design duration” in Table 3). This could be due to researchers’ apprehensions about device fatigue [165] and stakeholders’ concerns regarding privacy [166].

In the “ethics” reporting dimension, we explored factors such as consent models and monetary benefits because they are relevant to the likelihood of participants to share data. For example, consent models, as noticed in a survey conducted by Köngeter et al [167], have different acceptance rates among patients with cancer. In our scoping review, we found no publication reported blanket or meta consent (“Consent type” in Table 3). Since no studies collected data purely for secondary analysis (“Purpose of data collection” in Table 3), the preference for nonblanket consent was justified. Despite debates about meta consent [168] and dynamic consent [49] as solutions for modern research, our finding that no studies used meta (dynamic) consent aligns with critics who posit that these innovative approaches require highly participatory technological platforms and may introduce bias due to participants’ technical competence [169]. Over one-third of the original research publications that reported on monetary benefits provided no monetary benefits to participants (35%, 6/17; “Monetary benefits” in Table 3). Research generally indicates that even small monetary incentives increase consent and response rates [170], irrespective of risk level [171]. However, the impact of monetary benefits on wearable study participation remains undetermined and requires examination on a case-by-case basis (eg, for studies conducted on Amazon Mechanical Turk [9]) given the diversity of mobile device studies.

#### Prevalence of Reporting Deficiencies in Original Research Publications

Our results show a high level of reporting deficiencies in the “data” and “ethics” dimensions (Figure 4), with 5 reporting factors missing in over half of the original research publications—data ownership (91%, 41/45), participant access to data after data contribution (84%, 38/45), right to opt out (80%, 36/45), consent type (78%, 35/45), and monetary benefits (62%, 28/45). Among these 5 factors, we also noticed a trend over time of significantly declined performance in the reporting of data ownership (40% missingness, 2010-2016 vs 98% missingness, 2017-2021; Figure 5) and participant access to data after contribution (40% missingness, 2010-2016 vs 90% missingness, 2017-2021). However, over time, there was a significantly improved performance in reporting consent type (100% missingness, 2010-2016 vs 75% missingness, 2017-2021). In the “study” dimension, nearly 70% (31/45; Figure 4) of original research publications failed to report study participant attrition.

### Recommendations for Reporting Based on Findings From This Review

Our findings highlight several areas to consider when reporting research with PGHD collected using mobile devices. Recommendations for reporting based on findings from this review are summarized in Table 4. Further work is needed, however, to gain consensus on a more comprehensive set of reporting items. Such a consensus on best practices would greatly facilitate responsible and reproducible research and improve research quality and impact [172]. For example, a 2012 systematic review indicated a positive association between journal endorsements of a reporting guideline and the quality of reporting in randomized clinical trials published in those journals [173]. Another study looking at the publications from one journal found that those publications whose papers received editorial interventions designed to ensure adherence to reporting guidelines were more highly cited, with citation counts 43% higher than those publications that had not received the editorial interventions [174].

**Table 4:** Recommendations for reporting research with PGHD^a^ collected using mobile devices.

Reporting dimension	Reporting phenomena identified in this scoping review	Justification to include reporting dimension as a best practice
“Participant” (1 item)	“Purpose of data generation” item: Many publications reported the use of real-world PGHD from study participants (ie, not collected in controlled or experimental settings).	More detailed practices for correcting data quality issues that can arise with real-world PGHD should be documented so that others pursuing similar research can learn what works and does not work.
“Device” (2 items)	“Device owner” item: Many papers reported using a device owned by participants. “Device type” item: Many publications reported using wearables as secondary devices (ie, used with smartphones instead of as standalone devices).	The practice of using participant-owned devices should be monitored closely as there is potential for disparities in study participation for some groups due to a lack of device access. More details on the data transfer process should be given in study designs. It influences the ability to participate in research and the types of data that can be collected.
“Data” (2 items)	“Data owner” and “participant access to data after contribution” items: Publications largely lack descriptions of data ownership and often fail to describe how study participants can access their shared data.	If strategies for empowering study participants to maintain data ownership are not documented, studies cannot be performed to measure the effectiveness of these strategies or point to new approaches.
“Study” (1 item)	“Attrition (reported)” item: Attrition is often not reported.	Attrition can introduce selection bias and reduce statistical power due to data missingness [35]. Attrition should be documented so that others can evaluate the quality and fairness of data analyses.
“Ethics” (3 items)	“Consent type,” “right to opt out (reported),” and “monetary benefits” items: Descriptions of consent types, the rights of participants to opt out, and monetary benefits (if provided) are largely missing from publications.	Options provided to participants at the time of recruitment may influence their willingness to participate in research [167,170] and thereby influence the composition of the study population. Such options also inform the ethical assessment of a study.

^a^PGHD: person-generated health data.

### Limitations

Our review has limitations related to how we gathered evidence from the literature, our choice of reporting factors, and one of our data analysis methods.

Our PubMed search strategy (Textbox 1) has concepts with explicit data donation and data sharing terms to retrieve publications that describe participant data contributions. Because of this design, the search strategy only retrieved publications whose titles and abstracts had data donation and data sharing terms. Possibly, our search missed relevant publications that described participant data contributions only in the full text. The articles we analyzed were only collected from PubMed. Articles were not retrieved from other databases (eg, Embase or Web of Science), from the reference lists of included articles, or from the gray literature. Thus, it is possible that articles only available through these other sources were overlooked. Our preliminary searches, however, suggested that PubMed provided literature coverage sufficiently comprehensive for our research objectives.

While we selected reporting factors relevant to our goal of reviewing research that collects PGHD using mobile devices with the perspective of an individual’s data contribution, we may have excluded relevant factors. For example, we did not include the demographic factors of study populations, which may be more broadly relevant to wearable PGHD research. Detecting the low representation of some groups when using wearables for PGHD collection can indicate where biases exist that could lead to downstream inequities. This need is apparent based on findings that aging populations were underrepresented in studies with wearables [175] and that studies of cardiovascular disease that involve wearables [175]. Such a need requires domain-specific examinations, and we regard it out of the scope of this review, which aims to explore the landscape as the first step. As an example of a second-step effort, we have investigated the studies of cardiovascular disease that involve wearables, and we noticed that they did not reflect the demographics of patient populations in terms of age, race, education, cigarette smoking status, and hypertension status [152].

We lowered the granularity of some factors to avoid issues of imbalance in categorical analysis. For example, when analyzing research scenarios, we consolidated academic institutions, for-profit commercial companies, corporations or foundations, independent research organizations, patient-led groups, citizen scientists, and so on, into 3 categories. Thus, our findings do not necessarily represent the full range of possible ways of describing this research.

Despite these limitations, we are confident that having used strict procedures to reduce bias (eg, 2-level screening, independent charting, and a predefined charting template), this review offers a solid foundation for future research. We also feel that the full PubMed query we provide (Textbox 1) is a valuable contribution to other researchers since it was developed and tested for wearables and smartphones and since publications in the emerging field of mobile devices and PGHD are not well indexed.

### Conclusions

We identified trends and patterns in the reporting of research that collects PGHD using mobile devices. Given the growing interest in using a BYOD model among researchers, there is an opportunity for a broader use of crowdsourcing platforms for data transfer from participant-owned devices. There is also the opportunity to develop best practices that address observed reporting deficiencies in this research and make it more reproducible and responsible. Based on our findings, we recommend 9 items for enhanced reporting.

## Appendix

Multimedia Appendix 1 PRISMA-ScR (Preferred Reporting Items for Systematic Reviews and Meta-Analyses extension for Scoping Reviews) checklist.

Multimedia Appendix 2 Bibliographic information of the articles included in the scoping review.

Multimedia Appendix 3 Publication types across publication years and publication locations.

Multimedia Appendix 4 Reporting deficiencies in original research publications over time, as measured by the missingness of reporting factors.

## Abbreviations

BYOD: Bring-Your-Own-Device
FHIR: Fast Health Interoperability Resources
mERA: Mobile Health Evidence Reporting and Assessment
MeSH: medical subject headings
PGHD: person-generated health data
PRISMA-ScR: Preferred Reporting Items for Systematic Reviews and Meta-Analyses extension for Scoping reviews
STaRT-RWE: Structured Template and Reporting Tool for Real-World Evidence

## Data Availability

The data underlying this article are readily available upon reasonable request to the corresponding author.
