# Supplementary material for: Participant Contributions to Person-Generated Health Data Research Using Mobile Devices: Scoping Review
Source: J Med Internet Res. 2025 Jan 20;27:e51955. doi: 10.2196/51955 (PMC11791458; doi:10.2196/51955)
Supplement: Multimedia Appendix 2 [file jmir_v27i1e51955_app2.doc]

**Multimedia Appendix 2**. Bibliographic information of the articles included in the scoping review.

| Author | Bibliographic Dimension | | |
| --- | --- | --- | --- |
|  | Publication Year | Publication Location | Publication Type |
| Ajana et al. [1] | 2017 | Multiple | Other |
| Arab et al. [2] | 2010 | United States | Research |
| Atreja et al. [3] | 2015 | United States | Protocol |
| Auffray et al. [4] | 2016 | European Union | Other |
| Augusto et al. [5] | 2021 | Multiple | Other |
| Bietz et al. [6] | 2016 | United States | Research |
| Bloem et al. [7] | 2019 | European Union | Research |
| Bocher et al. [8] | 2017 | European Union | Tech |
| Boker et al. [9] | 2015 | United States | Tech |
| Bouras et al. [10] | 2020 | United States | Research |
| Bruno et al. [11] | 2021 | United Kingdom | Review |
| Buoite Stella et al. [12] | 2021 | European Union | Research |
| Burchert et al. [13] | 2021 | European Union | Research |
| Burkhardt et al. [14] | 2021 | United States | Tech |
| Cafazzo et al. [15] | 2012 | Canada | Research |
| Çelik Ertuğrul et al. [16] | 2021 | Other | Tech |
| Chan et al. [17] | 2018 | United States | Research |
| Chang et al. [18] | 2020 | United States | Research |
| Chen et al. [19] | 2016 | Other | Research |
| ChEuropean Unionng et al. [20] | 2016 | United States | Research |
| Chung et al. [21] | 2016 | United States | Tech |
| Clarke et al. [22] | 2021 | United Kingdom | Research |
| Cooke Bailey et al. [23] | 2018 | United States | Research |
| Cornet et al. [24] | 2018 | United States | Review |
| Deering et al. [25] | 2019 | United States | Tech |
| Deering et al. [26] | 2020 | United States | Tech |
| Eicher-Miller et al. [27] | 2021 | United States | Other |
| Evans et al. [28] | 2020 | United States | Other |
| Fadda et al. [29] | 2018 | Other | Protocol |
| Fadrique et al. [30] | 2020 | Canada | Review |
| Faurholt-Jepsen et al. [31] | 2019 | European Union | Research |
| Fish et al. [32] | 2021 | United Kingdom | Research |
| Franklin et al. [33] | 2020 | United States | Research |
| Freifeld et al. [34] | 2010 | United States | Review |
| Fylan et al. [35] | 2018 | United Kingdom | Research |
| Genes et al. [36] | 2018 | United States | Tech |
| Hafen et al. [37] | 2014 | Other | Other |
| Hamed et al. [38] | 2019 | United States | Research |
| Hartmann et al. [39] | 2019 | European Union | Research |
| Heidel et al. [40] | 2020 | European Union | Review |
| Heidel et al. [41] | 2021 | European Union | Research |
| Henderson et al. [42] | 2019 | United States | Protocol |
| Henriksen et al. [43] | 2021 | Other | Tech |
| Herbec et al. [44] | 2018 | United Kingdom | Research |
| Hershman et al. [45] | 2019 | United States | Tech |
| Hesse et al. [46] | 2016 | United States | Review |
| Hong et al. [47] | 2021 | Other | Research |
| Househ et al. [48] | 2018 | Other | Review |
| Hughes et al. [49] | 2016 | United Kingdom | Tech |
| Katapally et al. [50] | 2020 | Canada | Other |
| Kim et al. [51] | 2017 | Other | Protocol |
| Kim et al. [52] | 2019 | Other | Research |
| Kim et al. [53] | 2019 | Other | Research |
| Kolovson et al. [54] | 2020 | United States | Research |
| Kostkova et al. [55] | 2016 | United Kingdom | Review |
| Labs et al. [56] | 2020 | United States | Other |
| Laurie et al. [57] | 2019 | United Kingdom | Other |
| Leal Neto et al. [58] | 2017 | Other | Tech |
| Levin et al. [59] | 2021 | United States | Research |
| McKendry et al. [60] | 2020 | United Kingdom | Other |
| Moore et al. [61] | 2017 | United States | Review |
| Nakamoto et al. [62] | 2020 | Other | Tech |
| O’Doherty et al. [63] | 2016 | Canada | Review |
| Poletti et al. [64] | 2017 | European Union | Research |
| Poudyal et al. [65] | 2019 | United States | Protocol |
| Quer et al. [66] | 2021 | United States | Research |
| Radin et al. [67] | 2020 | United States | Research |
| Rake et al. [68] | 2017 | European Union | Other |
| Rendina et al. [69] | 2018 | United States | Research |
| Rieger et al. [70] | 2019 | United States | Research |
| Rohlman et al. [71] | 2019 | United States | Tech |
| Rothstein et al. [72] | 2015 | United States | Other |
| Ságvári et al. [73] | 2021 | European Union | Research |
| Salamone et al. [74] | 2021 | European Union | Review |
| Saleheen et al. [75] | 2016 | United States | Tech |
| Santos-Lozano et al. [76] | 2018 | European Union | Other |
| Schmitz et al. [77] | 2018 | United States | Review |
| Seltzer et al. [78] | 2019 | United States | Research |
| Simblett et al. [79] | 2020 | United Kingdom | Research |
| Sleigh et al. [80] | 2018 | European Union | Research |
| Slotwiner et al. [81] | 2019 | United States | Other |
| Smith et al. [82] | 2016 | United States | Other |
| Staccini et al. [83] | 2017 | European Union | Review |
| Struminskaya et al. [84] | 2020 | European Union | Research |
| Tajiri et al. [85] | 2018 | Other | Research |
| Tannis et al. [86] | 2019 | United States | Research |
| Taylor et al. [87] | 2020 | United States | Tech |
| van Heerden et al. [88] | 2020 | Other | Research |
| van Oeveren et al. [89] | 2019 | European Union | Research |
| Vitak et al. [90] | 2020 | United States | Other |
| Vo et al. [91] | 2019 | United States | Tech |
| von Gablenz et al. [92] | 2021 | European Union | Research |
| Wanyua et al. [93] | 2013 | Other | Tech |
| Wasfi et al. [94] | 2021 | Canada | Research |
| Webster et al. [95] | 2017 | United States | Tech |
| Welsh et al. [96] | 2019 | United States | Research |
| Wicks et al. [97] | 2018 | United States | Other |
| Witteveen et al. [98] | 2021 | United Kingdom | Research |
| Woldaregay et al. [99] | 2020 | European Union | Research |
| Yan et al. [100] | 2014 | Canada | Tech |

Bibliography

1. Ajana B. Digital health and the biopolitics of the Quantified Self. Digit Health 2017;**3**:2055207616689509 doi: 10.1177/2055207616689509.

2. Arab L, Winter A. Automated camera-phone experience with the frequency of imaging necessary to capture diet. J Am Diet Assoc 2010;**110**(8):1238-41 doi: 10.1016/j.jada.2010.05.010.

3. Atreja A, Khan S, Rogers JD, et al. Impact of the Mobile HealthPROMISE Platform on the Quality of Care and Quality of Life in Patients With Inflammatory Bowel Disease: Study Protocol of a Pragmatic Randomized Controlled Trial. JMIR Res Protoc 2015;**4**(1):e23 doi: 10.2196/resprot.4042.

4. Auffray C, Balling R, Barroso I, et al. Making sense of big data in health research: Towards an EUROPEAN UNION action plan. Genome Med 2016;**8**(1):71 doi: 10.1186/s13073-016-0323-y.

5. Augusto DG, Yusufali T, Peyser ND, et al. HLA-B*15:01 is associated with asymptomatic SARS-CoV-2 infection. medRxiv 2021 doi: 10.1101/2021.05.13.21257065.

6. Bietz MJ, Bloss CS, Calvert S, et al. Opportunities and challenges in the use of personal health data for health research. J Am Med Inform Assoc 2016;**23**(e1):e42-8 doi: 10.1093/jamia/ocv118 [published Online First: 2015/09/04].

7. Bloem BR, Marks WJ, Jr., Silva de Lima AL, et al. The Personalized Parkinson Project: examining disease progression through broad biomarkers in early Parkinson's disease. BMC NEuropean Unionrol 2019;**19**(1):160 doi: 10.1186/s12883-019-1394-3 [published Online First: 2019/07/19].

8. Bocher E, Petit G, Picaut J, et al. Collaborative noise data collected from smartphones. Data Brief 2017;**14**:498-503 doi: 10.1016/j.dib.2017.07.039.

9. Boker SM, Brick TR, Pritikin JN, et al. Maintained Individual Data Distributed Likelihood Estimation (MIDDLE). Multivariate Behav Res 2015;**50**(6):706-20 doi: 10.1080/00273171.2015.1094387.

10. Bouras A, Simoes EJ, Boren S, et al. Non-Hispanic White Mothers' Willingness to Share Personal Health Data With Researchers: Survey Results From an Opt-in Panel. J Particip Med 2020;**12**(2):e14062 doi: 10.2196/14062.

11. Bruno E, Böttcher S, Viana PF, et al. Wearable devices for seizure detection: Practical experiences and recommendations from the Wearables for Epilepsy And Research (WEAR) International Study Group. Epilepsia 2021;**62**(10):2307-21 doi: 10.1111/epi.17044.

12. Buoite Stella A, AjČeviĆ M, Furlanis G, et al. Smart technology for physical activity and health assessment during COVID-19 lockdown. J Sports Med Phys Fitness 2021;**61**(3):452-60 doi: 10.23736/S0022-4707.20.11373-2.

13. Burchert S, Kerber A, Zimmermann J, et al. Screening accuracy of a 14-day smartphone ambulatory assessment of depression symptoms and mood dynamics in a general population sample: Comparison with the PHQ-9 depression screening. PLoS One 2021;**16**(1):e0244955 doi: 10.1371/journal.pone.0244955.

14. Burkhardt HA, Brandt PS, Lee JR, et al. StayHome: A FHIR-Native Mobile COVID-19 Symptom Tracker and Public Health Reporting Tool. Online J Public Health Inform 2021;**13**(1):e2 doi: 10.5210/ojphi.v13i1.11462.

15. Cafazzo JA, Casselman M, Hamming N, et al. Design of an mHealth app for the self-management of adolescent type 1 diabetes: a pilot study. J Med Internet Res 2012;**14**(3):e70 doi: 10.2196/jmir.2058.

16. Çelik Ertuğrul D, Çelik Ulusoy D. A knowledge-based self-pre-diagnosis system to predict Covid-19 in smartphone users using personal data and observed symptoms. Expert Syst 2021 doi: 10.1111/exsy.12716.

17. Chan YY, Bot BM, Zweig M, et al. The asthma mobile health study, smartphone data collected using ResearchKit. Sci Data 2018;**5**:180096 doi: 10.1038/sdata.2018.96.

18. Chang AR, Bailey-Davis L, Hetherington V, et al. Remote Dietary Counseling Using Smartphone Applications in Patients With Stages 1-3a Chronic Kidney Disease: A Mixed Methods Feasibility Study. J Ren Nutr 2020;**30**(1):53-60 doi: 10.1053/j.jrn.2019.03.080.

19. Chen J, Bauman A, Allman-Farinelli M. A Study to Determine the Most Popular Lifestyle Smartphone Applications and Willingness of the Public to Share Their Personal Data for Health Research. Telemed J E Health 2016;**22**(8):655-65 doi: 10.1089/tmj.2015.0159 [published Online First: 2016/03/10].

20. ChEuropean Unionng C, Bietz MJ, Patrick K, et al. Privacy Attitudes among Early Adopters of Emerging Health Technologies. PLoS One 2016;**11**(11):e0166389 doi: 10.1371/journal.pone.0166389.

21. Chung AE, Sandler RS, Long MD, et al. Harnessing person-generated health data to accelerate patient-centered outcomes research: the Crohn's and Colitis Foundation of America PCORnet Patient Powered Research Network (CCFA Partners). J Am Med Inform Assoc 2016;**23**(3):485-90 doi: 10.1093/jamia/ocv191.

22. Clarke H, Clark S, Birkin M, et al. Understanding Barriers to Novel Data Linkages: Topic Modeling of the Results of the LifeInfo Survey. J Med Internet Res 2021;**23**(5):e24236 doi: 10.2196/24236.

23. Cooke Bailey JN, Crawford DC, Goldenberg A, et al. Willingness to Participate in a National Precision Medicine Cohort: Attitudes of Chronic Kidney Disease Patients at a Cleveland Public Hospital. J Pers Med 2018;**8**(3) doi: 10.3390/jpm8030021.

24. Cornet VP, Holden RJ. Systematic review of smartphone-based passive sensing for health and wellbeing. J Biomed Inform 2018;**77**:120-32 doi: 10.1016/j.jbi.2017.12.008.

25. Deering S, Grade MM, Uppal JK, et al. Accelerating Research With Technology: Rapid Recruitment for a Large-Scale Web-Based Sleep Study. JMIR Res Protoc 2019;**8**(1):e10974 doi: 10.2196/10974 [published Online First: 2019/01/22].

26. Deering S, Pratap A, Suver C, et al. Real-world longitudinal data collected from the SleepHealth mobile app study. Sci Data 2020;**7**(1):418 doi: 10.1038/s41597-020-00753-2 [published Online First: 2020/11/29].

27. Eicher-Miller HA, Prapkree L, Palacios C. Expanding the Capabilities of Nutrition Research and Health Promotion Through Mobile-Based Applications. Adv Nutr 2021;**12**(3):1032-41 doi: 10.1093/advances/nmab022.

28. Evans BJ. The Perils of Parity: Should Citizen Science and Traditional Research Follow the Same Ethical and Privacy Principles? J Law Med Ethics 2020;**48**(1_suppl):74-81 doi: 10.1177/1073110520917031.

29. Fadda M, Jobin A, Blasimme A, et al. User Perspectives of a Web-Based Data-Sharing Platform (Open Humans) on Ethical Oversight in Participant-Led Research: Protocol for a Quantitative Study. JMIR Res Protoc 2018;**7**(11):e10939 doi: 10.2196/10939.

30. Fadrique LX, Rahman D, Vaillancourt H, et al. Overview of Policies, Guidelines, and Standards for Active Assisted Living Data Exchange: Thematic Analysis. JMIR Mhealth Uhealth 2020;**8**(6):e15923 doi: 10.2196/15923 [published Online First: 2020/06/23].

31. Faurholt-Jepsen M, Þórarinsdóttir H, Vinberg M, et al. Automatically generated smartphone data and subjective stress in healthy individuals - a pilot study. Nord J Psychiatry 2020;**74**(4):293-300 doi: 10.1080/08039488.2019.1705904.

32. Fish LA, Jones EJH. A survey on the attitudes of parents with young children on in-home monitoring technologies and study designs for infant research. PLoS One 2021;**16**(2):e0245793 doi: 10.1371/journal.pone.0245793 [published Online First: 2021/02/06].

33. Franklin EF, Nichols HM, House L, et al. Cancer Patient Perspectives on Sharing of Medical Records and Mobile Device Data for Research Purposes. J Patient Exp 2020;**7**(6):1115-21 doi: 10.1177/2374373520923837.

34. Freifeld CC, Chunara R, Mekaru SR, et al. Participatory epidemiology: use of mobile phones for community-based health reporting. PLoS Med 2010;**7**(12):e1000376 doi: 10.1371/journal.pmed.1000376.

35. Fylan F, Caveney L, Cartwright A, et al. Making it work for me: beliefs about making a personal health record relevant and useable. BMC Health Serv Res 2018;**18**(1):445 doi: 10.1186/s12913-018-3254-z.

36. Genes N, Violante S, Cetrangol C, et al. From smartphone to EHR: a case report on integrating patient-generated health data. NPJ Digit Med 2018;**1**:23 doi: 10.1038/s41746-018-0030-8.

37. Hafen E, Kossmann D, Brand A. Health data cooperatives - citizen empowerment. Methods Inf Med 2014;**53**(2):82-6 doi: 10.3414/ME13-02-0051.

38. Hamed A, Curran C, Gwaltney C, et al. Mobility assessment using wearable technology in patients with late-onset Pompe disease. NPJ Digit Med 2019;**2**:70 doi: 10.1038/s41746-019-0143-8 [published Online First: 2019/07/26].

39. Hartmann R, Sander C, Lorenz N, et al. Utilization of Patient-Generated Data Collected Through Mobile Devices: Insights From a Survey on Attitudes Toward Mobile Self-Monitoring and Self-Management Apps for Depression. JMIR Ment Health 2019;**6**(4):e11671 doi: 10.2196/11671.

40. Heidel A, Hagist C. Potential Benefits and Risks Resulting From the Introduction of Health Apps and Wearables Into the German Statutory Health Care System: Scoping Review. JMIR Mhealth Uhealth 2020;**8**(9):e16444 doi: 10.2196/16444 [published Online First: 2020/09/24].

41. Heidel A, Hagist C, Schlereth C. Pricing through health apps generated data-Digital dividend as a game changer: Discrete choice experiment. PLoS One 2021;**16**(7):e0254786 doi: 10.1371/journal.pone.0254786 [published Online First: 2021/07/27].

42. Henderson ML, Thomas AG, Eno AK, et al. The Impact of the mKidney mHealth System on Live Donor Follow-Up Compliance: Protocol for a Randomized Controlled Trial. JMIR Res Protoc 2019;**8**(1):e11000 doi: 10.2196/11000.

43. Henriksen A, Johannessen E, Hartvigsen G, et al. Consumer-Based Activity Trackers as a Tool for Physical Activity Monitoring in Epidemiological Studies During the COVID-19 Pandemic: Development and United Statesbility Study. JMIR Public Health Surveill 2021;**7**(4):e23806 doi: 10.2196/23806 [published Online First: 2021/04/13].

44. Herbec A, Brown J, Shahab L, et al. Lessons learned from unsuccessful use of personal carbon monoxide monitors to remotely assess abstinence in a pragmatic trial of a smartphone stop smoking app - A secondary analysis. Addict Behav Rep 2019;**9**:100122 doi: 10.1016/j.abrep.2018.07.003.

45. Hershman SG, Bot BM, Shcherbina A, et al. Physical activity, sleep and cardiovascular health data for 50,000 individuals from the MyHeart Counts Study. Sci Data 2019;**6**(1):24 doi: 10.1038/s41597-019-0016-7.

46. Hesse BW, Greenberg AJ, Rutten LJ. The role of Internet resources in clinical oncology: promises and challenges. Nat Rev Clin Oncol 2016;**13**(12):767-76 doi: 10.1038/nrclinonc.2016.78.

47. Hong SJ, Cho H. Privacy Management and Health Information Sharing via Contact Tracing during the COVID-19 Pandemic: A Hypothetical Study on AI-Based Technologies. Health Commun 2021:1-12 doi: 10.1080/10410236.2021.1981565.

48. Househ M, Grainger R, Petersen C, et al. Balancing Between Privacy and Patient Needs for Health Information in the Age of Participatory Health and Social Media: A Scoping Review. Yearb Med Inform 2018;**27**(1):29-36 doi: 10.1055/s-0038-1641197.

49. Hughes A, Landers D, Arkenau HT, et al. Development and Evaluation of a New Technological Way of Engaging Patients and Enhancing Understanding of Drug Tolerability in Early Clinical Development: PROACT. Adv Ther 2016;**33**(6):1012-24 doi: 10.1007/s12325-016-0335-4.

50. Katapally TR. A Global Digital Citizen Science Policy to Tackle Pandemics Like COVID-19. J Med Internet Res 2020;**22**(5):e19357 doi: 10.2196/19357.

51. Kim G, Bae JC, Yi BK, et al. An information and communication technology-based centralized clinical trial to determine the efficacy and safety of insulin dose adjustment education based on a smartphone personal health record application: a randomized controlled trial. BMC Med Inform Decis Mak 2017;**17**(1):109 doi: 10.1186/s12911-017-0507-4.

52. Kim JW, Ryu B, Cho S, et al. Impact of Personal Health Records and Wearables on Health Outcomes and Patient Response: Three-Arm Randomized Controlled Trial. JMIR Mhealth Uhealth 2019;**7**(1):e12070 doi: 10.2196/12070.

53. Kim TK, Choi M. Older adults' willingness to share their personal and health information when adopting healthcare technology and services. Int J Med Inform 2019;**126**:86-94 doi: 10.1016/j.ijmedinf.2019.03.010.

54. Kolovson S, Pratap A, Duffy J, et al. Understanding Participant Needs for Engagement and Attitudes towards Passive Sensing in Remote Digital Health Studies. Int Conf Pervasive Comput Technol Healthc 2020;**2020**:347-62 doi: 10.1145/3421937.3422025.

55. Kostkova P, Brewer H, de Lusignan S, et al. Who Owns the Data? Open Data for Healthcare. Front Public Health 2016;**4**:7 doi: 10.3389/fpubh.2016.00007.

56. Labs J, Terry S. Privacy in the Coronavirus Era. Genet Test Mol Biomarkers 2020;**24**(9):535-36 doi: 10.1089/gtmb.2020.29055.sjt.

57. Laurie GT. Cross-Sectoral Big Data: The Application of an Ethics Framework for Big Data in Health and Research. Asian Bioeth Rev 2019;**11**(3):327-39 doi: 10.1007/s41649-019-00093-3.

58. Leal Neto O, Dimech GS, Libel M, et al. Saúde na Copa: The World's First Application of Participatory Surveillance for a Mass Gathering at FIFA World Cup 2014, Brazil. JMIR Public Health Surveill 2017;**3**(2):e26 doi: 10.2196/publichealth.7313.

59. Levin HI, Egger D, Andres L, et al. Sensing everyday activity: Parent perceptions and feasibility. Infant Behav Dev 2021;**62**:101511 doi: 10.1016/j.infbeh.2020.101511 [published Online First: 2021/01/20].

60. McKendry RA, Rees G, Cox IJ, et al. Share mobile and social-media data to curb COVID-19. Nature 2020;**580**(7801):29 doi: 10.1038/d41586-020-00908-6.

61. Moore S, Tassé AM, Thorogood A, et al. Consent Processes for Mobile App Mediated Research: Systematic Review. JMIR Mhealth Uhealth 2017;**5**(8):e126 doi: 10.2196/mhealth.7014 [published Online First: 2017/09/01].

62. Nakamoto I, Jiang M, Zhang J, et al. Evaluation of the Design and Implementation of a Peer-To-Peer COVID-19 Contact Tracing Mobile App (COCOA) in Japan. JMIR Mhealth Uhealth 2020;**8**(12):e22098 doi: 10.2196/22098.

63. O'Doherty KC, Christofides E, Yen J, et al. If you build it, they will come: unintended future uses of organised health data collections. BMC Med Ethics 2016;**17**(1):54 doi: 10.1186/s12910-016-0137-x.

64. Poletti P, Visintainer R, Lepri B, et al. The interplay between individual social behavior and clinical symptoms in small clustered groups. BMC Infect Dis 2017;**17**(1):521 doi: 10.1186/s12879-017-2623-2 [published Online First: 2017/07/28].

65. Poudyal A, van Heerden A, Hagaman A, et al. Wearable Digital Sensors to Identify Risks of Postpartum Depression and Personalize Psychological Treatment for Adolescent Mothers: Protocol for a Mixed Methods Exploratory Study in Rural Nepal. JMIR Res Protoc 2019;**8**(8):e14734 doi: 10.2196/14734.

66. Quer G, Gadaleta M, Radin JM, et al. The Physiologic Response to COVID-19 Vaccination. medRxiv 2021 doi: 10.1101/2021.05.03.21256482.

67. Radin JM, Peters S, Ariniello L, et al. Pregnancy health in POWERMOM participants living in rural versus urban zip codes. J Clin Transl Sci 2020;**4**(5):457-62 doi: 10.1017/cts.2020.33.

68. Rake EA, van Gelder M, Grim DC, et al. Personalized Consent Flow in Contemporary Data Sharing for Medical Research: A Viewpoint. Biomed Res Int 2017;**2017**:7147212 doi: 10.1155/2017/7147212 [published Online First: 2017/06/24].

69. Rendina HJ, Mustanski B. Privacy, Trust, and Data Sharing in Web-Based and Mobile Research: Participant Perspectives in a Large Nationwide Sample of Men Who Have Sex With Men in the United States. J Med Internet Res 2018;**20**(7):e233 doi: 10.2196/jmir.9019.

70. Rieger A, Gaines A, Barnett I, et al. Psychiatry Outpatients' Willingness to Share Social Media Posts and Smartphone Data for Research and Clinical Purposes: Survey Study. JMIR Form Res 2019;**3**(3):e14329 doi: 10.2196/14329.

71. Rohlman D, Dixon HM, Kincl L, et al. Development of an environmental health tool linking chemical exposures, physical location and lung function. BMC Public Health 2019;**19**(1):854 doi: 10.1186/s12889-019-7217-z.

72. Rothstein MA, Wilbanks JT, Brothers KB. Citizen Science on Your Smartphone: An ELSI Research Agenda. J Law Med Ethics 2015;**43**(4):897-903 doi: 10.1111/jlme.12327.

73. Ságvári B, Gulyás A, Koltai J. Attitudes towards Participation in a Passive Data Collection Experiment. Sensors (Basel) 2021;**21**(18) doi: 10.3390/s21186085.

74. Salamone F, Masullo M, Sibilio S. Wearable Devices for Environmental Monitoring in the Built Environment: A Systematic Review. Sensors (Basel) 2021;**21**(14) doi: 10.3390/s21144727.

75. Saleheen N, Chakraborty S, Ali N, et al. mSieve: Differential Behavioral Privacy in Time Series of Mobile Sensor Data. Proc ACM Int Conf Ubiquitous Comput 2016;**2016**:706-17 doi: 10.1145/2971648.2971753.

76. Santos-Lozano A, Baladrón C, Martín-Hernández J, et al. mHealth and the legacy of John Snow. Lancet 2018;**391**(10129):1479-80 doi: 10.1016/S0140-6736(18)30783-9.

77. Schmitz H, Howe CL, Armstrong DG, et al. Leveraging mobile health applications for biomedical research and citizen science: a scoping review. J Am Med Inform Assoc 2018;**25**(12):1685-95 doi: 10.1093/jamia/ocy130.

78. Seltzer E, Goldshear J, GuntUnited Kingdomu SC, et al. Patients' willingness to share digital health and non-health data for research: a cross-sectional study. BMC Med Inform Decis Mak 2019;**19**(1):157 doi: 10.1186/s12911-019-0886-9 [published Online First: 2019/08/10].

79. Simblett SK, Biondi A, Bruno E, et al. Patients' experience of wearing multimodal sensor devices intended to detect epileptic seizures: A qualitative analysis. Epilepsy Behav 2020;**102**:106717 doi: 10.1016/j.yebeh.2019.106717.

80. Sleigh J. Experiences of Donating Personal Data to Mental Health Research: An Explorative Anthropological Study. Biomed Inform Insights 2018;**10**:1178222618785131 doi: 10.1177/1178222618785131 [published Online First: 2018/07/18].

81. Slotwiner DJ, Tarakji KG, Al-Khatib SM, et al. Transparent sharing of digital health data: A call to action. Heart Rhythm 2019;**16**(9):e95-e106 doi: 10.1016/j.hrthm.2019.04.042.

82. Smith RJ, Grande D, Merchant RM. Transforming Scientific Inquiry: Tapping Into Digital Data by Building a Culture of Transparency and Consent. Acad Med 2016;**91**(4):469-72 doi: 10.1097/ACM.0000000000001022.

83. Staccini P, Fernandez-Luque L. Secondary Use of Recorded or Self-expressed Personal Data: Consumer Health Informatics and Education in the Era of Social Media and Health Apps. Yearb Med Inform 2017;**26**(1):172-77 doi: 10.15265/iy-2017-037 [published Online First: 2017/10/25].

84. Struminskaya B, Toepoel V, Lugtig P, et al. Understanding Willingness to Share Smartphone-Sensor Data. Public Opin Q 2020;**84**(3):725-59 doi: 10.1093/poq/nfaa044 [published Online First: 2021/05/25].

85. Tajiri E, Yoshimura E, Hatamoto Y, et al. Effect of sleep curtailment on dietary behavior and physical activity: A randomized crossover trial. Physiol Behav 2018;**184**:60-67 doi: 10.1016/j.physbeh.2017.11.008.

86. Tannis C, Senerat A, Garg M, et al. Improving Physical Activity among Residents of Affordable Housing: Is Active Design Enough? Int J Environ Res Public Health 2019;**16**(1) doi: 10.3390/ijerph16010151.

87. Taylor S, Jaques N, Nosakhare E, et al. Personalized Multitask Learning for Predicting Tomorrow's Mood, Stress, and Health. IEEE Trans Affect Comput 2020;**11**(2):200-13 doi: 10.1109/TAFFC.2017.2784832.

88. van Heerden A, Wassenaar D, Essack Z, et al. In-Home Passive Sensor Data Collection and Its Implications for Social Media Research: Perspectives of Community Women in Rural South Africa. J Empir Res Hum Res Ethics 2020;**15**(1-2):97-107 doi: 10.1177/1556264619881334.

89. Van Oeveren BT, De Ruiter CJ, Hoozemans MJM, et al. Inter-individual differences in stride frequencies during running obtained from wearable data. J Sports Sci 2019;**37**(17):1996-2006 doi: 10.1080/02640414.2019.1614137.

90. Vitak J, Zimmer M. More Than Just Privacy: Using Contextual Integrity to Evaluate the Long-Term Risks from COVID-19 Surveillance Technologies. Soc Media Soc 2020;**6**(3):2056305120948250 doi: 10.1177/2056305120948250.

91. Vo JDV, Gorbach AM. A Platform to Record Patient Events During Physiological Monitoring With Wearable Sensors: Proof-of-Concept Study. Interact J Med Res 2019;**8**(1):e10336 doi: 10.2196/10336.

92. von Gablenz P, Kowalk U, Bitzer J, et al. Individual Hearing Aid Benefit in Real Life Evaluated Using Ecological Momentary Assessment. Trends Hear 2021;**25**:2331216521990288 doi: 10.1177/2331216521990288.

93. Wanyua S, Ndemwa M, Goto K, et al. Profile: the Mbita health and demographic surveillance system. Int J Epidemiol 2013;**42**(6):1678-85 doi: 10.1093/ije/dyt180.

94. Wasfi R, Poirier Stephens Z, Sones M, et al. Recruiting Participants for Population Health Intervention Research: Effectiveness and Costs of Recruitment Methods for the INTERACT (INTErventions, Research, and Action in Cities Team) study. J Med Internet Res 2021 doi: 10.2196/21142.

95. Webster DE, Suver C, Doerr M, et al. The Mole Mapper Study, mobile phone skin imaging and melanoma risk data collected using ResearchKit. Sci Data 2017;**4**:170005 doi: 10.1038/sdata.2017.5.

96. Welsh JB, Derdzinski M, Parker AS, et al. Real-Time Sharing and Following of Continuous Glucose Monitoring Data in Youth. Diabetes Ther 2019;**10**(2):751-55 doi: 10.1007/s13300-019-0571-0.

97. Wicks P. Patient, study thyself. BMC Med 2018;**16**(1):217 doi: 10.1186/s12916-018-1216-2.

98. Witteveen D, de Pedraza P. The Roles of General Health and COVID-19 Proximity in Contact Tracing App United Statesge: Cross-sectional Survey Study. JMIR Public Health Surveill 2021;**7**(8):e27892 doi: 10.2196/27892.

99. Woldaregay AZ, Henriksen A, Issom DZ, et al. User Expectations and Willingness to Share Self-Collected Health Data. Stud Health Technol Inform 2020;**270**:894-98 doi: 10.3233/SHTI200290.

100. Yan K, Tracie B, Marie-Ève M, et al. Innovation through Wearable Sensors to Collect Real-Life Data among Pediatric Patients with Cardiometabolic Risk Factors. Int J Pediatr 2014;**2014**:328076 doi: 10.1155/2014/328076.
