# Supplementary material for: Participant Contributions to Person-Generated Health Data Research Using Mobile Devices: Scoping Review
Source: J Med Internet Res. 2025 Jan 20;27:e51955. doi: 10.2196/51955 (PMC11791458; doi:10.2196/51955)
Supplement: Multimedia Appendix 3 [file jmir_v27i1e51955_app3.doc]

**Multimedia Appendix 3**. Publication types across publication years and publication locations.

|  | | Research | Protocol | Tech | Review | Other | Overall |
| --- | --- | --- | --- | --- | --- | --- | --- |
|  |  | N=45 (45%) | N=5 (5%) | N=20 (20%) | N=13 (13%) | N=17 (17%) | N=100 |
| Publication year | | | | | | | |
|  | 2010-2016 | 5 (11%) | 1 (20%) | 6 (30.0%) | 4 (33%) | 4 (24%) | 20 (20%) |
|  | 2017-2021 | 40 (89%) | 4 (80%) | 14 (70.0%) | 9 (69%) | 13 (77%) | 80 (80%) |
| Publication location | | | | | | | |
|  | United States | 18 (40%) | 3 (60%) | 12 (60.0%) | 5 (39%) | 8 (47%) | 46 (46%) |
|  | United Kingdom | 6 (13%) | 0 (0%) | 1 (5.0%) | 2 (15%) | 2 (12%) | 11 (11%) |
|  | European Union | 13 (29%) | 0 (0%) | 1 (5.0%) | 3 (23%) | 3 (18%) | 20 (20%) |
|  | Canada | 2 (4%) | 0 (0%) | 1 (5.0%) | 2 (15%) | 1 (6%) | 6 (6%) |
|  | Multiple | 0 (0%) | 0 (0%) | 0 (0%) | 0 (0%) | 2 (12%) | 2 (2%) |
|  | Other | 6 (13%) | 2 (40%) | 5 (25.0%) | 1 (8%) | 1 (6%) | 15 (15%) |
