# Supplementary material for: Participant Contributions to Person-Generated Health Data Research Using Mobile Devices: Scoping Review
Source: J Med Internet Res. 2025 Jan 20;27:e51955. doi: 10.2196/51955 (PMC11791458; doi:10.2196/51955)
Supplement: Multimedia Appendix 4 [file jmir_v27i1e51955_app4.doc]

**Multimedia Appendix 4**. Reporting deficiencies in original research publications over time, as measured by the missingness of reporting factors.

| **Reporting Factors** | | **Proportions of publications having missingness; n(missing)/n(total), %** | | |
| --- | --- | --- | --- | --- |
|  |  | 2010-2016 | 2017-2021 | Change of % |
| *Participant Dimension* | | | | |
|  | IdentityDataSubjectAndContributor | 0/5, 0.0% | 4/40, 10.0% | 10.0% |
|  | LivingStatus | 0/5, 0.0% | 1/4, 2.5% | 2.5% |
|  | PurposeDataGeneration | 2/5, 40.0% | 13/40, 32.5% | -7.5% |
| *Device Dimension* | | | | |
|  | DeviceOwner | 2/5, 40.0% | 15/40, 37.5% | -2.5% |
|  | DeviceType | 1/5, 20.0% | 0/40, 0.0% | -20.0% |
|  | DataCaptureMode | 2/5, 40.0% | 5/40, 12.5% | -27.5% |
| *Data Dimension* | | | | |
|  | DataType | 0/5, 0.0% | 0/40, 0.0% | 0.0% |
|  | DataOwner | 2/5, 40.0% | 39/40, 97.5% | 57.5% |
|  | DataAccess | 2/5, 40.0% | 36/40, 90.0% | 50.0% |
| *Study Dimension* | | | | |
|  | ResearchScenario | 0/5, 0.0% | 2/40, 5.0% | 5.0% |
|  | PurposeDataCollection | 1/5, 20.0% | 14/40, 35.0% | 15.0% |
|  | CrowdsourcingPlatform | 5/5, 100.0% | 35/40, 87.5% | -12.5% |
|  | DesignObsExp | 0/5, 0.0% | 0/40, 0.0% | 0.0% |
|  | DesignDuration | 0/5, 0.0% | 2/40, 5.0% | 5.0% |
|  | Attrition | 3/5, 60.0% | 28/40, 70.0% | 10.0% |
| *Ethics Dimension* | | | | |
|  | MonetaryBenefits | 3/5, 60.0% | 25/40, 62.5% | 2.5% |
|  | Consent | 0/5, 0.0% | 4/40, 10.0% | 10.0% |
|  | ConsentInformed | 2/5, 40.0% | 15/40, 37.5% | -2.5% |
|  | ConsentType | 5/5, 100.0% | 30/40, 75.0% | -25.0% |
|  | ConsentSubject | 2/5, 40.0% | 6/40, 15.0% | -25.0% |
|  | RightOptOut | 4/5, 80.0% | 32/40, 80.0% | 0.0% |
